# The efficacy of the simple bypass protocol for chronic cerebral arterial occlusion and moyamoya disease

**DOI:** 10.1186/s41016-025-00413-7

**Published:** 2025-10-27

**Authors:** Ittipon Gunnarut, Kritsada Buakate

**Affiliations:** Neurosurgical Unit, Department of Surgery, Rajavithi Hospital, College of Medicine, Rangsit University, Bangkok, 10400 Thailand

**Keywords:** Chronic cerebral arterial occlusion, Mean transit time, Cerebral bypass

## Abstract

**Background:**

Ischemic strokes represent a significant public health concern, with a prevalence of 2.5% in the United States and over 250,000 new cases annually in Thailand, where strokes remain the leading cause of mortality. Chronic cerebral arterial occlusion and moyamoya disease are specific subtypes of ischemic stroke. In certain regions, advanced diagnostic tools are often inaccessible. Simple bypass protocols, which utilize mean transit time (MTT) in conjunction with surgical interventions such as the single-barrel bypass, are valuable for enhancing patient outcomes in these settings. The objective of this study is to elucidate the efficacy of MTT as a diagnostic tool and to evaluate the single-barrel bypass as a therapeutic intervention for chronic cerebral arterial occlusion.

**Methods:**

This retrospective study assessed the utility of MTT as a selection criterion and evaluated the efficacy of the single-barrel bypass procedure for chronic cerebral arterial occlusion. Conducted at Rajavithi Hospital, the study included patients treated between 2010 and 2024 with complete medical records. Outcomes measured were changes in neurological function, alterations in MTT, and the incidence of surgical complications within one month postoperatively.

**Results:**

Among the 30 patients who underwent the simple bypass protocol, 80% (24/30) demonstrated symptomatic improvement and better Modified Rankin Scale scores. Only one complication was reported—a brainstem infarction in a single patient.

**Conclusions:**

The simple bypass protocol is an effective intervention for patients exhibiting prolonged MTT and is particularly useful in regions where stress tests such as acetazolamide or CO2 challenge testing are unavailable.

## Background

Ischemic strokes represent a major global health burden, particularly in Thailand, where strokes are the leading cause of death and disability. Chronic cerebral arterial occlusion contributes significantly to ischemic stroke incidence, leading to prolonged reductions in cerebral blood flow and subsequent neurological deficits. According to the 2020 American Heart Association report Heart Disease and Stroke Statistics, the prevalence of strokes in adults in the United States in 2016 was approximately 2.5% [1]. Strokes are the greatest cause of mortality and disability in Thailand, with an estimated 250,000 new cases reported each year, claiming around 50,000 lives. Alarming statistics from the Thai Ministry of Public Health rank strokes as the most common cause of death among both men and women. Large vessel occlusion ischemic strokes account for about 31% of all stroke cases [2]. Chronic cerebral arterial occlusion, one of the causes of ischemic stroke, leads to sustained reductions in blood flow and fosters an ischemic environment that precipitates neurological deficits. In the early 2000 s, therapy for chronic arterial occlusion primarily consisted of conservative treatment with medication. However, the limitations of drug therapy alone are now better understood. Even with more intensive drug treatment, studies report that the annual incidence of recurrent strokes remains as high as 15% [3–6].

Historically, management of chronic arterial occlusion primarily relied on conservative drug therapies, but these have shown limited efficacy, with studies reporting an annual recurrence rate of 15% despite intensive pharmacological treatment. Current therapeutic options include medical management, cerebral bypass surgery, and endovascular procedures. However, patient selection for surgical intervention remains a controversial issue. For moyamoya disease, characterized by progressive stenosis of cerebral arteries, revascularization surgery is often necessary. However, the appropriate selection of patients for bypass surgery remains a crucial aspect of treatment. There is a critical need for effective diagnostic and therapeutic strategies to mitigate the adverse outcomes of this condition. One of the key requirements for understanding and managing chronic cerebral arterial occlusion or moyamoya disease is the accurate selection of patients suitable for bypass surgery. Various imaging methods are available to assist in identifying appropriate candidates for cerebral bypass, but each technique has its limitations. Mean transit time (MTT), derived from perfusion imaging, has emerged as a critical diagnostic tool, offering insights into cerebral hemodynamics. Prolonged MTT suggests compromised cerebrovascular reserve and serves as a potential indicator for surgical intervention. This study evaluates the efficacy of MTT in identifying suitable surgical candidates and assesses the outcomes of single-barrel bypass surgery in these patients [7–9].

Surgical interventions, such as the single- or double-barrel bypass microsurgical techniques, involve anastomosing a branch of the external carotid artery to a cortical branch of a cerebral artery. These procedures aim to enhance cerebral blood flow and reduce the risk of ischemic events. Successful outcomes depend on meticulous planning and execution, with effectiveness often evaluated through postoperative clinical improvements.

The choice between single- and double-barrel bypass remains a topic of debate [10, 11]. The objective of this journal article is to elucidate the efficacy of mean transit time as a diagnostic tool and to evaluate the single-barrel bypass as a therapeutic intervention for chronic cerebral arterial occlusion. By analyzing recent clinical data and exploring advanced imaging techniques, we aim to provide a comprehensive understanding of how these modalities can be integrated into clinical practice to enhance patient management and outcomes. Through this exploration, the potential to refine diagnostic accuracy and improve surgical success rates in addressing chronic cerebral ischemia will be highlighted.

## Materials and methods

This retrospective study aimed to evaluate the efficacy of mean transit time (MTT) as a selection tool and to appraise the single-barrel bypass as a therapeutic intervention in patients with chronic cerebral arterial occlusion. Patient data were anonymized to ensure confidentiality. All procedures were performed in accordance with the ethical standards of the institutional and national research committees, as well as the 1964 Helsinki Declaration, including its later amendments or comparable ethical standards. For this type of study, formal consent was not required. Ethical approval was obtained from the Ethics Committee of Rajavithi Hospital (No. 189/2567).

**Fig. 1:**
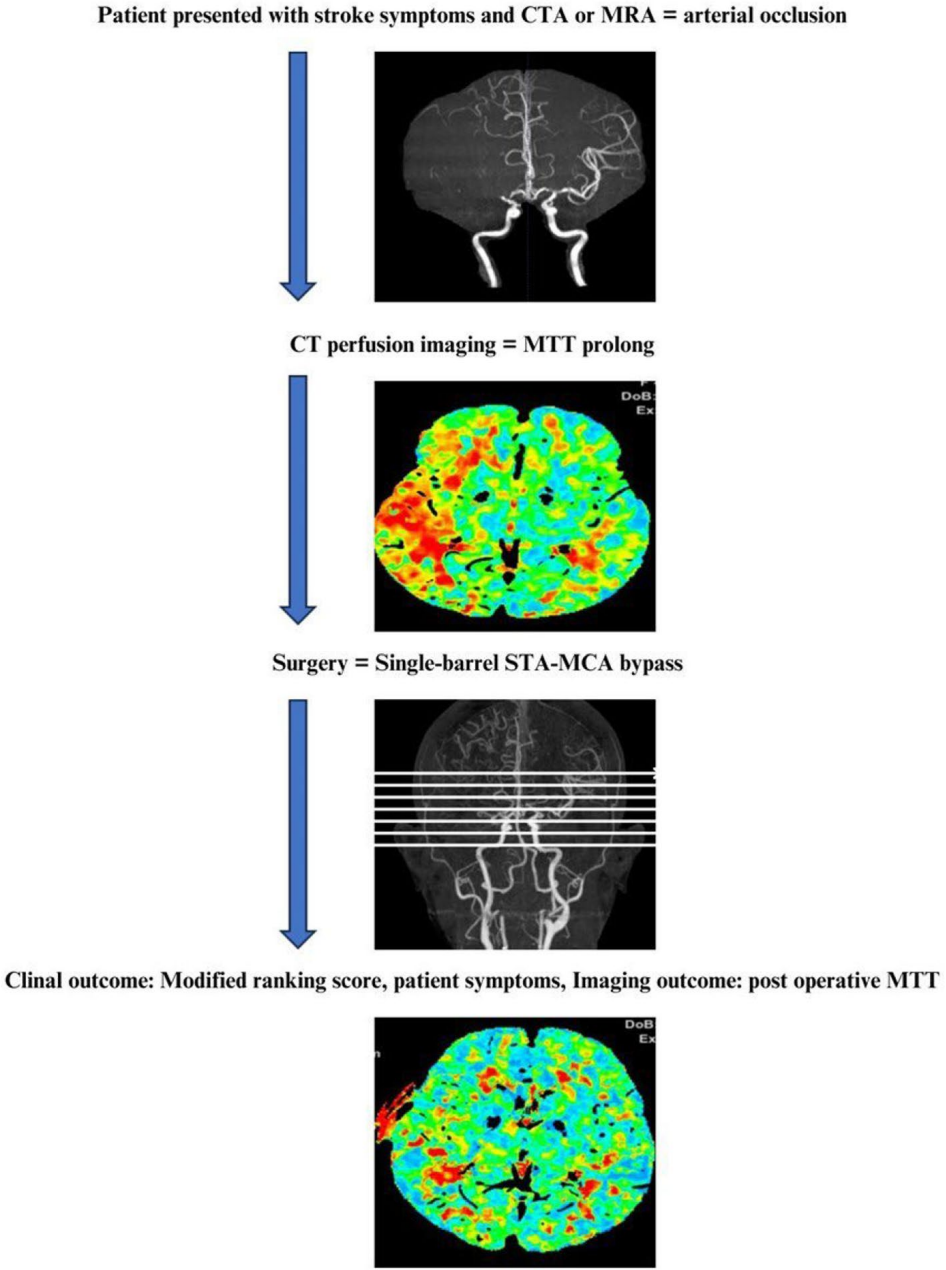
Protocol for simple bypass

Between January 2010 and December 2024, patients who underwent single-barrel bypass surgery for chronic cerebral occlusion or Moyamoya disease, diagnosed by cerebrovascular imaging, were enrolled if they had complete preoperative perfusion imaging and postoperative follow-up records for at least one month. All patients were performed with simple bypass protocol as Fig. 1.

For patients with chronic cerebral occlusion, inclusion criteria were: (1) treatment with dual antiplatelet therapy and (2) the presence of ischemic symptoms. For patients with Moyamoya disease, indications included: (1) evidence of cerebral occlusion or bleeding, (2) infarctions smaller than 2 cm or resolved hemorrhagic lesions, (3) Suzuki stage 2–4 on angiography, and (4) a post-event interval of more than two months.

The threshold for hemodynamic compromise was defined as mean transit time (MTT) prolongation of more than 6 s [12]. CT perfusion was performed using a GE LightSpeed VCT (64-slice) scanner in cine mode (8 × 5 mm, 4 cm coverage) with the following parameters: 80 kVp, 200 mAs, rotation time 0.5 s, matrix 512 × 512, and field of view 220–240 mm. Images were acquired continuously for 55 s, beginning approximately 5 s after contrast injection (iohexol 350–370 mgI/mL, 45 mL at 4 mL/s), followed by a 30 mL saline flush at 4 mL/s.

Post-processing was conducted on a GE Advantage Workstation version 4.7 using the CT/Brain Perfusion 4D module. A deconvolution algorithm with delay correction and motion correction enabled was applied. The arterial input function was placed in the contralateral ACA A2 segment, and the venous output function was set in the superior sagittal sinus. Cases with bolus failure (arterial input function peak < 80 HU) or motion artifacts greater than 2 mm were excluded.

Patients were excluded if they had incomplete medical records, experienced an acute stroke, suffered from a severe comorbid condition affecting cerebral hemodynamics, or were pregnant or lactating during the study period.

Patient data were retrospectively collected from hospital databases and medical records. Demographic information included age, gender, and relevant medical history. Clinical evaluations were based on baseline and follow-up neurological assessments recorded in medical records, which were conducted according to patient symptoms and their Modified Rankin Scores (mRS). Pre-surgical perfusion imaging studies were reviewed to obtain MTT values using CT perfusion. Data regarding the single-barrel bypass procedure were also collected, including vessel selection, intraoperative findings, and any complications.

Primary Outcome: Changes in neurological function scores (GCS and mRS).

Secondary Outcomes: Changes in MTT at presentation, incidence of adverse events and surgical complications, and documented clinical symptom improvement during follow-up visits.

Statistical analysis was performed using SPSS software version 22.0 (SPSS Inc., Chicago, Illinois, USA). Continuous data for demographic and clinical characteristics were presented as means ± standard deviations, while categorical data were expressed as counts and percentages.

## Results

A total of 30 chronic cerebral arterial occlusion patients from Rajavithi Hospital were enrolled. Table 1 shows the demographic and clinical characteristics of the subjects. Two-thirds of the participants were male, and moyamoya patients accounted for one-third of the cases. MTT delay on both sides was observed in 23.3% of cases, and the mean pre-operative Modified Rankin score was 2.50. Table 2 shows postoperative outcomes and complications. Postoperative symptoms improved in 80% of cases, with only one case of complication. Intraoperative graft patency was good, at 93.3%.

**Table 1:** Demographic and clinical characteristics of the study patients (*n* = 30)

Characteristics	*n* (%)
Gender	
Male	20 (66.7)
Female	10 (33.3)
Underlining disease	13 (43.3)
Diagnostics	
Moyamoya disease	10 (33.3)
Others	20 (66.7)
Graft type	
Single	29 (96.7)
Other	1 (3.3)
CT perfusion MTT	
Delay right side	9 (30.0)
Delay left side	14 (46.7)
Delay both sides	7 (23.3)
CT perfusion CBV	
Normal	22 (73.3)
Decreased	3 (10.0)
Increased	5 (16.7)
Pre-operative symptoms GSC	
15	26 (86.7)
Others	4 (13.3)
Pre-operative mRS (Mean ± SD)	2.50 ± 1.64
Median (min–max)	2.5 (0.0–5.0)

Table 1 summarizes the demographic and baseline clinical data of the enrolled patients. The majority were male. Moyamoya disease was diagnosed in 10 patients (33.3%). Single-barrel bypass was performed in 29 patients (96.7%), while one patient (3.3%) underwent interpositional bypass. Preoperative Glasgow Coma Scale (GCS) scores were 15 in 26 patients (86.7%), and the mean preoperative Modified Rankin Scale (mRS) score was 2.5

**Table 2:** Postoperative outcomes and complications

Characteristics	Total (*n* = 30) *n* (%)
Post-operative symptoms GSC	
15	29 (96.7)
Others	1 (3.3)
Post-operative symptoms CC	
Improved	24 (80.0)
Same	6 (20.0)
Post-operative mRS (Mean ± SD)	1.79 ± 1.37
Median (min–max)	2.0 (0.0–4.0)
Intra-operative graft patency	
Good	28 (93.3)
Failed	2 (6.7)
Post-operative CTA	
Good	28 (93.3)
Failed	2 (6.7)
CT perfusion MTT	
No post operative CT perfusion	21 (70.0)
Improved	7 (23.3)
Same	2 (6.7)
CT perfusion CBV	
No	21 (70.0)
Same	9 (30.0)
Follow-up time (Mean ± SD)	10.10 ± 7.26
Median (min–max)	9 (1–36)
Post operative complications	
No	29 (96.7)
Brain stem infarction	1 (3.3)

Table 2 presents the postoperative outcomes. Clinical symptoms improved in 24 patients (80.0%). Postoperative CT perfusion was performed in 9 patients (30.0%), of whom 7 patients (23.3%) demonstrated improvement in mean transit time (MTT), while 2 patients (6.7%) showed no significant change. The remaining 21 patients (70.0%) did not undergo postoperative CT perfusion. Notably, all patients who underwent postoperative CT perfusion regardless of MTT changes experienced clinical improvement

The postoperative Glasgow Coma Scale (GCS) score was 15 in 29 patients (96.7%), and the mean postoperative Modified Rankin Scale (mRS) score was 2.0

## Examples of patients that underwent simple bypass protocol

### First patient

**Fig. 2:**
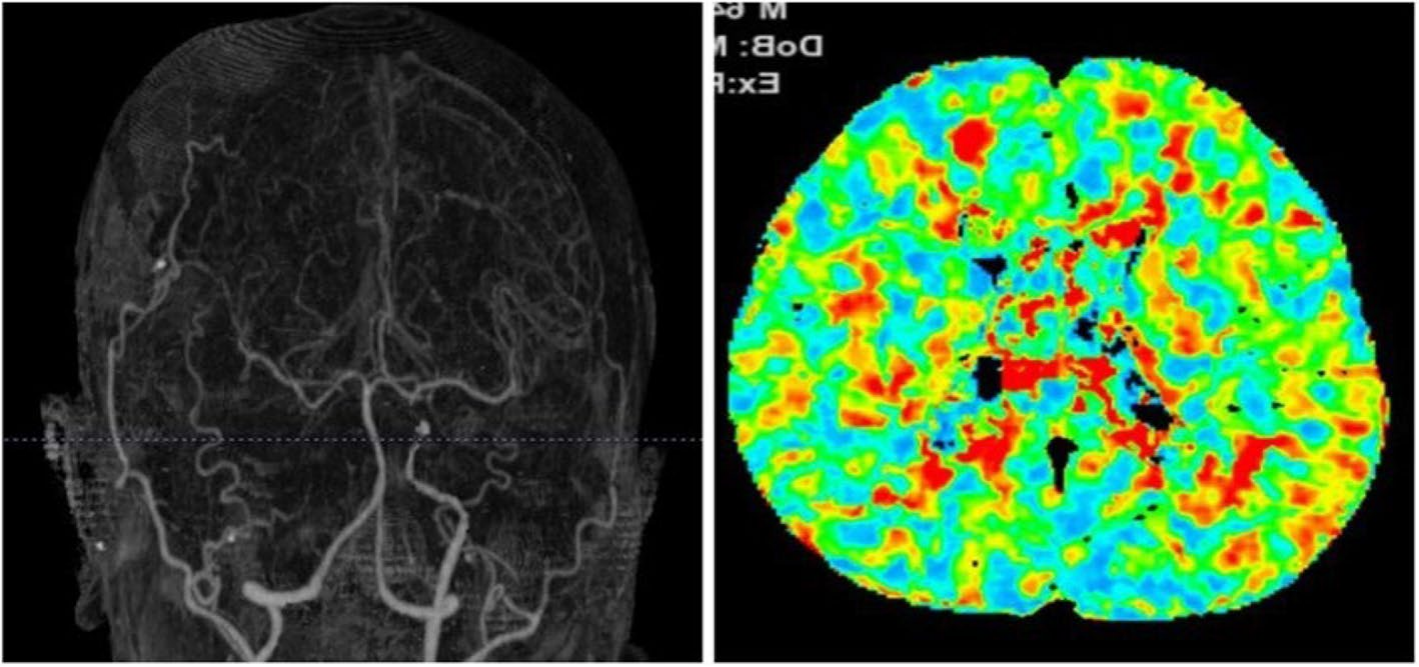
CTA and CT perfusion

A Thai male patient presented with TIA GSC 15, (right eye blindness), motor power grade 5 at all. Pre-op imaging shows right ICA occlusion with prolonged MTT on both sides (Fig. 2).

**Fig. 3:**
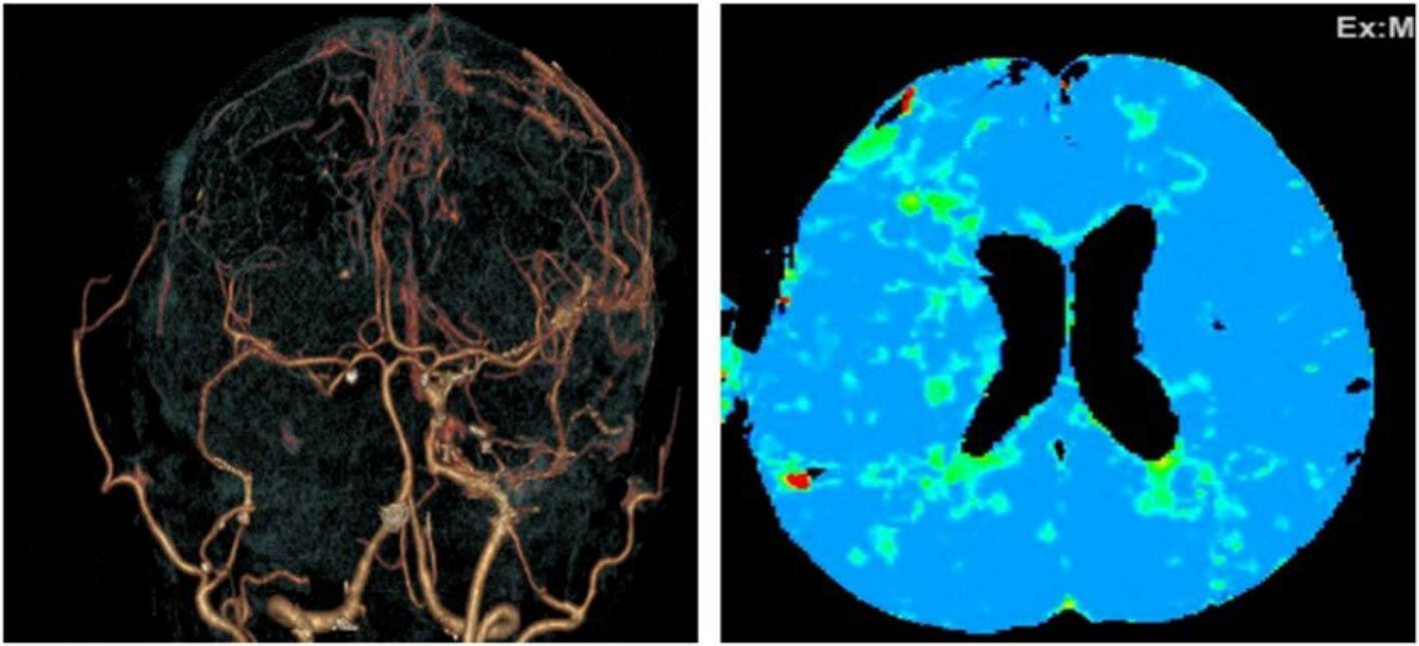
Post-operative CTA and CT perfusion

This patient underwent right single-barrel STA-MCA bypass after which his post-operative symptoms improved, and there was no TIA. CTA and CT perfusion showed improvement, as shown below (Fig. 3).

### Second patient

**Fig. 4:**
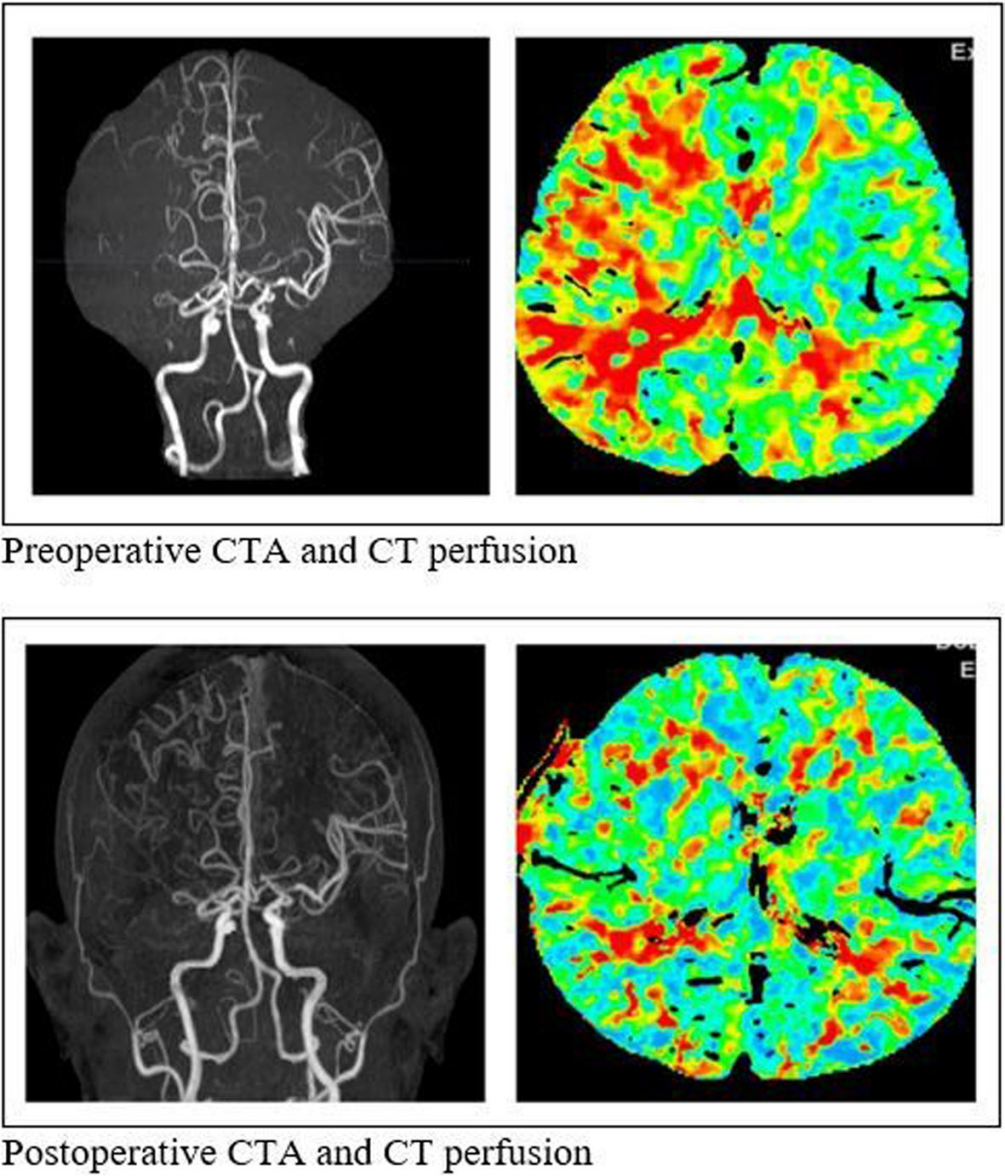
Pre-Postoperative CTA and CT perfusion

A Thai female presented with left-side weakness grade 4 +, GSC 15 on dual antiplatelet. After right STA-MCA bypass, the patient’s symptoms had improved: motor grade 5 at all and post-operative CTA and CT perfusion had improved (Fig. 4).

## Discussion

Ischemic strokes continue to present high morbidity and mortality rates worldwide. Chronic cerebral arterial occlusion exacerbates these outcomes, necessitating effective interventions. Cerebral reserve assessment is fundamental for determining surgical suitability, with two primary testing methods:

1. Stress Tests: Including acetazolamide or CO₂ challenge tests, though these carry risks of inducing ischemic events and may not be widely available in some regions.
2. Non-Stress Tests: Alternative methods such as MTT imaging, which is particularly useful in low-resource settings.

The stress test is a process used to assess the cerebral reserve. The disadvantage of this process is that it carries the risk of inducing neurological complications such as ischemic stroke and other issues. In some countries, such as Thailand, acetazolamide is not used.

The non-stress test protocol used for assessing a patient’s suitability for cerebral bypass is normally used in developing countries, as it does not present the risk of neurosurgical complications; furthermore, in low-income nations, the resources required for stress tests may not be available or affordable [13].

Although the Carotid Occlusion Surgery Study in the United States found that bypass surgery had no beneficial effects in patients with elevated oxygen extraction fraction (OEF) due to internal carotid artery (ICA) occlusion, the Japan EC-IC Bypass Trial demonstrated its effectiveness in patients with hemodynamic compromise [14, 15].

With regard to MTT and cerebrovascular reserve, as mean transit time is a compensatory condition of vasoreactivity, in which the cerebral blood flow is decreased and the cerebral blood volume increased, the mean transit time does not increase significantly. However, once the limit of vasoreactivity (maximal dilatation) has been reached, the mean transit time begins to rise. Therefore, the mean transit time should be more closely correlated with the percentage of cerebral vascular reserve [16–19].

Keiichi Kikuchi et al. [16] found that mean transit time images generated from dynamic susceptibility contrast-enhanced MR imaging data could be used to evaluate the extent of cerebral perfusion reserve impairment in patients with occlusive cerebrovascular disease. They showed that the mean transit time was correlated with cerebrovascular reserve.

Michael Mu Huo Teng et al. [17] concluded that quantitative results for the brain area with MTT prolongation were positively correlated with improvement in brain perfusion in MTT, EC-IC bypass patency, and patient outcomes.

Atsumu Hashimoto et al. [18] used the MTT as measured by CT perfusion in combination with CBF as measured at resting state. They reported that 123 I-IMP SPECT may be useful for evaluating hemodynamic compromise as an alternative to the acetazolamide challenge test.

Eunhee Kim et al. [19] concluded that mean transit time is the best predictive parameter for assessing decreased CVR in patients with unilateral steno-occlusive vascular disease.

As in the aforementioned study by Atsumu Hashimoto et al. [18] our research used MTT to evaluate CVR, and we found that all patients with prolonged MTT who underwent surgical bypass had better post-operative outcomes. We concluded that prolonged MTT is correlated with good clinical outcome and impaired vascular reserve. One patient one patient had brain stem infarction post-operatively, but this operative complication was is not involved in the bypass area.

### Single or double bypass

Some research papers in the literature, such as one by Jan-Karl Burkhardt et al. [20] have compared single and double-barrel bypass and have reported that DB STA-MCA bypasses should be reserved for patients requiring revascularization of multiple vascular territories or efferent arteries. DB STA-MCA bypasses have patency rates and patient outcomes comparable with those of SB STA-MCA, with the additional advantages of requiring only a single incision. They are also of lower operative complexity compared to high-flow bypasses and are therefore an important element in the vascular neurosurgeon's bypass armamentarium. Mahnjeong Ha et al. [21] used the single bypass in ischemic moyamoya disease and concluded that it achieved good outcomes. Xuying Chang [22] compared the results of STA-MCA using the single- and double-barrel techniques and found that the latter technique was superior in terms of cerebral blood flow and neurological outcomes. More recently, Huang C. et al. [10] performed comparative single- and double-bypass in moyamoya disease and concluded that although the double-barrel bypass could be used, it was no more effective than its single-branch alternative.

In this study, only single-barrel bypass procedures were performed, and favorable clinical outcomes were observed in all patients. Among the seven patients who underwent postoperative CT perfusion, all demonstrated improvements in perfusion parameters, such as mean transit time (MTT), along with resolution of their preoperative symptoms. The correlation between MTT reduction and postoperative symptomatic improvement was positive, consistent with previous studies showing that a significant decrease in MTT following surgery corresponds with clinical recovery [23].

Notably, all patients who underwent postoperative CT perfusion regardless of whether their MTT values improved—showed clinical improvement. This finding suggests that, although preoperative MTT prolongation remains a valuable criterion for patient selection, postoperative improvement in MTT is not the sole determinant of favorable outcomes. Other mechanisms, including enhanced collateral circulation and neurovascular adaptation, may also play a role in symptomatic recovery after bypass surgery [24].

In this paper we performed single bypasses only, and 80% of all patients had ameliorated clinical symptoms, and their post-operative Modified Rankin Score also improved, as shown in Table 2.

### Compare with another protocol

The Carotid Occlusion Surgery Study (COSS) reported that bypass surgery did not significantly benefit patients with elevated oxygen extraction fraction (OEF) due to ICA occlusion. Conversely, the Japanese EC-IC Bypass Trial demonstrated improved outcomes in patients with hemodynamic compromise. Recent publications have reported positive results in patients with chronic cerebral arterial occlusion. For example, Lars Wessels, MD, et al. [23] used O-15 H2O positron emission tomography (PET) or Single Photon Emission Computed Tomography (SPECT) during an acetazolamide (Diamox) challenge. They found that the patency rate of bypass grafts was greater than 90%, with a postoperative stroke rate of 4.3% and a one-year ischemic/TIA event rate of 6.1%. Cheng Qiu et al. [25] reported on cerebral bypass surgery in patients with chronic terminal internal carotid and/or middle cerebral artery occlusion, using early-arriving flow proportion (EFP) to evaluate cerebral blood flow (CBF). They found that a CBF ratio of 1.525 s on the lesion side to 2.525 s on the contralateral side correlated with better clinical outcomes in surgical patients compared to those receiving medical treatment alone. Postoperative complications occurred in approximately 11.76% of the surgical group. Keiichi Kikuchi et al. found MTT to be a reliable indicator of cerebral reserve impairment, correlating with postoperative improvements [26]. This study confirms that prolonged MTT correlates with impaired vascular reserve and supports its role in patient selection for bypass surgery. In this study, we used mean transit time (MTT) to evaluate cerebrovascular reactivity (CVR), and the clinical outcome improved in 80% of cases. Only one patient experienced a brainstem stroke one month after the operation.

## Conclusions

The simple bypass protocol demonstrates effectiveness in treating patients with chronic cerebral occlusion and moyamoya disease, particularly in resource-limited settings where stress tests are not available. The procedure has a high success rate, minimal complications, and offers a cost-effective alternative for evaluating cerebrovascular reactivity. Future research should focus on larger cohorts and direct comparisons between single- and double-barrel techniques.

## Limitation and future

This report has several limitations. First, its retrospective design inherently introduces the risk of selection bias, as patient inclusion was dependent on available records and referral practices. In our cohort, most patients presented with relatively severe hemodynamic compromise, prolonged MTT, or symptomatic chronic cerebral occlusion/moyamoya disease meeting surgical indications. Therefore, the study population may not fully represent patients with milder forms of disease who were managed conservatively. Second, there is a possibility of missing data, such as incomplete follow-up information or missing postoperative imaging, which could influence the accuracy of our outcome assessments. Third, potential confounding factors may have influenced both efficacy and safety outcomes. These include comorbidities such as hypertension, diabetes mellitus, and dyslipidemia, which are known to affect cerebrovascular outcomes; differences in preoperative medical management, since all patients were on dual antiplatelet therapy but the intensity and duration of therapy varied compared with other non-surgical cohorts; and variations in perioperative care, including surgeon experience, anesthetic management, and postoperative rehabilitation protocols, which may have differed across individual cases.

## Data Availability

The data supporting the results of this article are included within the article.

## Abbreviations

MTT: Mean transit time
GSC: Glasgow coma score
mRS: Modified Rank Scores
ICA: Internal carotid artery
EC-IC bypass: Extracranial-Intracranial bypass
STA-MCA bypass: Superficial temporal artery-middle cerebral artery bypass
CRV: Cerebrovascular reserve
